# *In Vitro* Antimicrobial Bioassays, DPPH Radical Scavenging Activity, and FTIR Spectroscopy Analysis of* Heliotropium bacciferum*

**DOI:** 10.1155/2016/3818945

**Published:** 2016-08-11

**Authors:** Sohail Ahmad, Naser M. AbdEl-Salam, Riaz Ullah

**Affiliations:** ^1^Department of Chemistry, Qurtuba University of Science and Information Technology Peshawar, Peshawar 25120, Pakistan; ^2^Riyadh Community College, King Saud University, Riyadh 11437, Saudi Arabia; ^3^Department of Chemistry, Government College Ara Khel, FR Kohat 26000, Pakistan

## Abstract

The present study deals with the antimicrobial, antioxidant, and functional group analysis of* Heliotropium bacciferum* extracts. Disc diffusion susceptibility method was followed for antimicrobial assessment. Noteworthy antimicrobial activities were recorded by various plant extracts against antibiotic resistant microorganisms. Plant flower extracts antioxidant activity was investigated against 2, 2-diphenyl-1-picryl hydrazyl radical by ultraviolet spectrophotometer (517 nm). Plant extracts displayed noteworthy radical scavenging activities at all concentrations (25–225 *μ*g/mL). Notable activities were recorded by crude, chloroform and ethyl acetate extracts up to 88.27% at 225 *μ*g/mL concentration. Compounds functional groups were examined by Fourier transform infrared spectroscopic studies. Alkanes, alkenes, alkyl halides, amines, carboxylic acids, amides, esters, alcohols, phenols, nitrocompounds, and aromatic compounds were identified by FTIR analysis. Thin layer chromatography bioautography was carried out for all plant extracts. Different bands were separated by various solvent systems. The results of the current study justify the use of* Heliotropium bacciferum* in traditional remedial herbal medicines.

## 1. Introduction

Plants are the foremost sources of traditional medicines with a huge variety of bioactive components, which are effective against various diseases. Plants biological activities are attributed to these bioactive components. Medicinal plants which are the rich sources of antifungal and antibacterial agents are used as basis of effective beneficial drugs in many countries [1]. Fungi, bacteria, viruses, and other microorganisms are potentially pathogenic to humans and animals. Worldwide, antibiotic resistant bacteria epidemics have been reported in hospitals. Therefore, discovery of novel antimicrobial agents to fight such diseases becomes very significant and indispensable [2]. Medicinal plants are the richest sources of these microbial agents. In traditional systems of modern medicines, plants are the richest resources of drugs, food supplements, folk medicines, nutraceuticals, and chemical permitted for synthetic drugs. Plant potential as source of novel drugs is still largely unfamiliar. In phytochemical perspective, only a slight percentage of plant has been explored [3]. Worldwide, antimicrobial agent's resistance has amplified and caused considerable mortality and morbidity. Bacteria have genetic capability to transmit and gain resistance to therapeutically active drugs. In developing countries, elevated cost of drug treatment has contributed to eminent frequency of opportunistic and chronic diseases. To manage these infections, there is an essential search for novel agents with less toxicity and larger antibacterial activity [4–7].* Heliotropium* genus belongs to Boraginaceae family consists of about 100 genera and 2000 species [8]. Flavonoids and polyphenols distribution in Boraginaceae family has different pharmaceutical activities such as antibacterial, antioxidant, anti-inflammatory, antiviral, and hepatoprotecting [9].* Heliotropium bacciferum* is a potent source of various phytochemicals and reported significant Diphenyl picryl hydrazyl (DPPH) radical scavenging activities [10]. It is a wealthy source of pyrrolizidine alkaloids, which have antimicrobial, antihyperlipidemic, antidiabetic, and antitumor properties [11]. Previously reported study revealed that the aerial parts of* Heliotropium bacciferum* have significant antibacterial and antifungal effects. All tested plant extracts exhibited significant activities against different bacterial and fungal strains. The result against various microorganisms divulged the curative potential of the plant* Heliotropium bacciferum* [12].

There is no reported data on the antimicrobial activities of individual parts of the plant. Therefore, the present research was designed to screen the antibacterial, antifungal (leaves, flowers, and stem), and antioxidant (flower) assays of* Heliotropium bacciferum *extracts. Investigation of bioactive compounds functional groups and thin layer chromatography bioautography was also the key focus of the present study.

## 2. Materials and Methods

### 2.1. Plant Collection and Authentication

*Heliotropium bacciferum* was collected from Karak, KPK, Pakistan. Sample washing and cleansing were accomplished by deionized water for further processing. Plant parts (leaves, flowers, and stem) were separated, dried at room temperature, and crushed into coarse powder. Herbarium staff of Plant Sciences, University of Peshawar, authenticates the plant species and kept the plant species in the laboratory for further processing.

### 2.2. Chemicals and Reagents

Analytical and HPLC grade chemicals and reagents were used for experimental screening. Methanol,* n*-hexane, chloroform, ethyl acetate, and* n*-butanol were used for plant constituent's extraction. Solvent purification was accomplished by dehydrating agents (Na_2_SO_4_ and MgSO_4_).

### 2.3. Plant Extraction and Fractionation

Plant parts, that is, leaves, flowers, and stem, were shade dried and pulverized into powder form. Maceration was carried out in methanol (CH_3_OH) for two weeks by Rehman et al. [13] methodology. Crude methanol extract of plant leaves (95 g), flowers (83 g), and stem (78 g) was suspended in distilled water (500 mL) and portioned in sequence with* n-*hexane (30 g, 22 g, and 18 g), chloroform (28 g, 25 g, and 20 g), ethyl acetate (34 g, 26 g, and 20 g), and* n-*butanol (31 g, 24 g, and 17 g), respectively.

### 2.4. Antibacterial Assay

Antibacterial assay of leaves, flowers, and stem extracts of* Heliotropium bacciferum* was investigated by disc diffusion susceptibility method [14]. Seven bacterial species, that is,* Escherichia coli* (ATCC 25922),* Staphylococcus aureus* (ATCC 6538)*, Bacillus cereus* (ATCC 7722)*, Pseudomonas aeruginosa* (ATCC 9721),* Klebsiella pneumoniae* (ATCC 6824)*, Proteus mirabilis* (ATCC 7103), and* Erwinia carotovora* (ATCC 8452) were used for antibacterial bioassay. The solvents used for antibacterial evaluation were purified by dehydrating agents such as Na_2_SO_4_ and MgSO_4_. Fractional distillation was also carried out for further solvent purification. Plant extract stock solutions (1 mg/mL) were prepared in dimethyl sulfoxide (DMSO). Nutrient agar media (2.8 g/100 mL) were used for microbe's culturing and growth, while nutrient broth (1.3 g/100 mL) was used for microorganism's standardization. Standardized microbial cultures (50 *μ*L) with glass spreader were inoculated on each nutrient agar plate in a laminar flow hood for microbial growth and incubated at 37°C for 24 hrs. The first streaked cultures were inoculated and incubated again. The second streaked microbial cultures were inoculated in nutrient broth (20–25 mL) and incubated for 18 hrs at 37°C in shaking water bath (200 rpm). Sterilized nutrient broth dilution was accomplished for standardization of microbial cultures and compared with 0.5 McFarland turbidity standard. Whatman filter paper discs (5 mm in diameter) were placed on solidified agar media with the help of sterilized forceps. Plant extracts (15 *μ*g/disc) of leaves, flower, and stem were applied to media plates and incubated at 37°C for 24 hours. As negative control, DMSO (5%) was used, while ampicillin antibiotic (8 *μ*g/disc) was used as a positive control. The zone of inhibition (mm) was then measured for each plant extract.

### 2.5. Antifungal Assay

Agar well diffusion technique was used for the assessment of antifungal bioassay. Five fungal strains, that is,* Aspergillus niger, Aspergillus flavus, Aspergillus parasiticus, Aspergillus oryzae, *and* Aspergillus fumigatus*, were used for this activity. Fungal strains were cultured on Sabouraud's dextrose agar (SDA) media for 3–5 days at 28°C. Nutrient broth media (28 g/1000 mL) were prepared in distilled water for the refreshment of fungal strains. Sterilized SDA plates were taken and 6 mm diameter sterile cork borer was used to bore wells in the agar media. Plant extracts (15 *μ*g/well) were then added into each well. Plates were allowed to stand for 1 hour at 37°C for extract diffusion into agar and incubated at 28°C for 24 hrs. DMSO (5%) was taken as negative control, while clotrimazole antibiotic (8 *μ*g/well) was used as positive control. The zone of inhibition (mm) was then measured for each plant extract.

### 2.6. DPPH Radical Scavenging Activity

Antioxidant activity of plant flower extracts was investigated against 2, 2-diphenyl-1-picryl hydrazyl radical by ultraviolet spectrophotometer (517 nm). The methodology of Ahmad et al. [10] was used for the activity. Plant extracts stock solution (1000 mg/mL) was prepared and diluted (25, 75, 125, 175, and 225 *μ*g/mL) with the respective solvents. Ascorbic acid was used as control for comparison. The same five dilutions were also prepared for control. DPPH solution was prepared at concentration of 0.003 g/100 mL. All plant extracts were then treated with DPPH solution. Spectrophotometer was used for absorbance calculation at 517 nm after 30 mints. Absorbance decline by DPPH solution was used as an indication for high antioxidant activity. The percent antioxidant activity was calculated by the given formula:

(1) $$\begin{array}{c} \%\;RSA={DPPH}_{Ab}-\frac{{Sample}_{Ab}}{{DPPH}_{Ab}}. \end{array}$$

### 2.7. Fourier Transform Infrared Spectroscopy (FTIR)

Functional groups and types of chemical bonds present in phytochemicals are identified by Fourier transform infrared spectroscopy analysis. Light absorbed wavelength is the prominent aspect of chemicals bonds, which can be seen through interpreted spectrum. Compound chemical bonds can be deduced via absorption infrared spectrum.* Heliotropium bacciferum* extracts (8 mg) were directed for FTIR assessment. Each plant extract (8 mg) was loaded to Fourier transform infrared spectrophotometer for functional group analysis. Functional group analysis was accomplished by Fourier transform infrared spectrophotometer (IRTracer-100, Shimadzu, Japan). Minute quantity of* Heliotropium bacciferum* flower extracts were placed on sample holder of FTIR at constant pressure. The IR peaks absorbance (wave number, cm^−1^) was recorded in the range of 4000 cm^−1^ to 400 cm^−1^.

### 2.8. Thin Layer Chromatography (TLC) Bioautography

Thin layer chromatography bioautography was accomplished by using EMW (ethyl acetate : methanol : water) (40 : 6 : 5), CEF (chloroform : ethyl acetate : formic acid) (9 : 7 : 2), and BEA (benzene/ethanol/ammonium hydroxide) (16 : 4 : 2) solvent systems of all plant extracts. Different bands were separated at various solvent systems. Ultraviolet light (305 and 368 nm) was used for the screening of those bands which were not visible in day light on TLC plates. Plant extracts (8 mg/mL) were applied on TLC plates in a fine band. Elution of these extracts was accomplished by three various solvent systems. For traces of removal of solvents, TLC plates were dried for five days under swift moving air. Bacterial new grown cultures were added on Mueller-Hinton broth. Bacterial strains densities used for* Escherichia coli*,* Staphylococcus aureus*,* Bacillus cereus*,* Pseudomonas aeruginosa*,* Klebsiella pneumoniae*,* Proteus mirabilis*, and* Erwinia carotovora* were approximately 3 × 10^2^, 2 × 10^4^, 2 × 10^7^, 3 × 10^3^, 2 × 10^5^, 3 × 10^6^, and 2 × 10^2^ cfu per milliliter, respectively. Freshly cultured fungal strains were added to Sabouraud dextrose broth. Fungal strains densities used for* Aspergillus niger*,* Aspergillus flavus*,* Aspergillus parasiticus*,* Aspergillus oryzae*, and* Aspergillus fumigatus* were about 5 × 10^5^, 3 × 10^4^, 3 × 10^6^, 2 × 10^3^, and 2 × 10^5^ cells per milliliter, respectively. Bacterial and fungal suspensions were sprayed on TLC chromatograms. Laminar flow hood was used for bacterial processing and biosafety cabinet was used for fungal processing. TLC plates were then kept in dark (100% relative humidity) at 35°C for a night. The plates were sprayed with p-iodonitrotetrazolium (2 mg/mL) violet and incubated overnight.

### 2.9. Statistical Analysis

All values were presented as the mean ± standard error of mean and analyzed for Two-Way ANOVA and One-Way ANOVA. Statistical analysis was carried out on GraphPad PRISM 6.

## 3. Results

### 3.1. Antibacterial Activity

The antibacterial activity of* Heliotropium bacciferum *leaves, flowers, and stem extracts was recorded against various microorganisms. All plant extracts exhibit a range of inhibitory potentials (Table 1). Methanol,* n-*hexane, and ethyl acetate extracts of plant leaves (15 *μ*g) revealed significant activities (18 ± 0.46 mm, 20 ± 0.71 mm, and 21 ± 0.69 mm) against* Klebsiella pneumoniae*,* Staphylococcus aureus* (16 ± 0.51 mm, 17 ± 0.34 mm, and 19 ± 0.53 mm),* Pseudomonas aeruginosa* (16 ± 0.44 mm, 17 ± 0.58 mm, and 15 ± 0.53 mm), and* Escherichia coli* (13 ± 0.32 mm, 19 ± 0.46 mm, and 18 ± 0.65 mm), respectively. Plant leaves chloroform and* n-*butanol extracts (15 *μ*g) were active against* Pseudomonas aeruginosa* (16 ± 0.37 mm and 14 ± 0.75 mm) and* Klebsiella pneumoniae* (17 ± 0.73 mm and 10 ± 0.28 mm) and were completely inactive against* Staphylococcus aureus* and* Erwinia carotovora*. Plant flowers* n-*hexane, ethyl acetate, and* n-*butanol extracts (15 *μ*g) showed prominent activities against* Escherichia coli* (17 ± 0.46 mm, 16 ± 0.64 mm, and 14 ± 0.34 mm),* Staphylococcus aureus* (19 ± 0.76 mm, 20 ± 0.74 mm, and 11 ± 0.54 mm), and* Klebsiella pneumoniae* (19 ± 0.75 mm, 19 ± 0.48 mm, and 13 ± 0.46 mm), respectively. Chloroform and* n-*butanol extracts (15 *μ*g) of plant stem showed noteworthy activities (15 ± 0.53 mm and 11 ± 0.43 mm) against* Escherichia coli* and* Klebsiella pneumonia* (17 ± 0.56 mm and 15 ± 0.64 mm), respectively. Aqueous extracts (15 *μ*g) of plant stem were active against* Klebsiella pneumoniae* (13 ± 0.42 mm)*, Proteus mirabilis* (10 ± 0.29 mm), and* Erwinia carotovora* (11 ± 0.26 mm). Ethyl acetate and* n-*hexane extracts (15 *μ*g) of plant stem were active against all bacterial microorganisms and revealed prominent activities in the range of 11–18 mm (Table 1).

### 3.2. Antifungal Activity

The antifungal activity of* Heliotropium bacciferum *leaves, flowers, and stem extracts was recorded against various fungal strains. All plant extracts exhibit a range of inhibitory potentials as shown in Table 2. Plant methanol,* n*-hexane, chloroform, ethyl acetate, and* n-*butanol extracts (15 *μ*g) of leaves showed prominent activities against* Aspergillus niger* (17 ± 0.44 mm, 14 ± 0.52 mm, 12 ± 0.28 mm, 15 ± 0.43 mm, and 11 ± 0.43 mm),* Aspergillus flavus* (15 ± 0.38 mm, 17 ± 0.67 mm, 13 ± 0.53 mm, 17 ± 0.32 mm, and 14 ± 0.51 mm), and* Aspergillus oryzae* (11 ± 0.54 mm, 16 ± 0.68 mm, 16 ± 0.45 mm, 17 ± 0.83 mm, and 15 ± 0.57 mm), respectively. Plant methanol, chloroform, and* n-*butanol extracts (15 *μ*g) of flowers revealed noteworthy activities against* Aspergillus niger* (14 ± 0.25 mm, 11 ± 0.26 mm, and 13 ± 0.47 mm) and* Aspergillus flavus* (17 ± 0.63 mm, 14 ± 0.46 mm, and 11 ± 0.23 mm), respectively. Significant activities were recorded by* n*-hexane and ethyl acetate extracts of plant flowers against* Aspergillus niger* (17 ± 0.63 mm and 16 ± 0.59 mm),* Aspergillus flavus* (15 ± 0.48 mm, 15 ± 0.59 mm), and* Aspergillus oryzae* (12 ± 0.27 mm and 15 ± 0.44 mm), respectively. Methanol and chloroform extracts (15 *μ*g) of plant stem were active against* Aspergillus niger* (16 ± 0.54 mm and 15 ± 0.54 mm) and* Aspergillus fumigatus* (15 ± 0.51 mm and 14 ± 0.37 mm), respectively. Excellent activities were shown by* n-*hexane and ethyl acetate extracts (15 *μ*g) against* Aspergillus flavus* (11 ± 0.31 mm and 18 ± 0.50 mm),* Aspergillus oryzae* (17 ± 0.54 mm and 15 ± 0.55 mm), and* Aspergillus fumigatus* (12 ± 0.33 mm and 16 ± 0.54 mm), respectively (Table 2).

### 3.3. DPPH Radical Scavenging Activity

*Heliotropium bacciferum* flower extracts revealed significant antioxidant activities which are shown in Table 3. Noteworthy activities (91.58%) were recorded by control (ascorbic acid) at 225 *μ*g/mL concentration. Plant crude extract exhibited excellent radical scavenging activities (70.12–85.75%) at all concentrations (25–225 *μ*g/mL). Notable activities were recorded for* n*-hexane and* n*-butanol in the range of 65.47–81.14% at various dilutions. Chloroform extract showed significant antioxidant activity up to 86.34% at 225 *μ*g/mL concentration. Ethyl acetate extract displayed excellent radical scavenging activities (71.27–88.43%) at all dilutions. Aqueous extracts (72.36%) were slightly active and revealed notable activity at higher concentrations (225 *μ*g/mL) (Table 3).

### 3.4. Fourier Transform Infrared Spectroscopy (FTIR)

**Figure 1:**
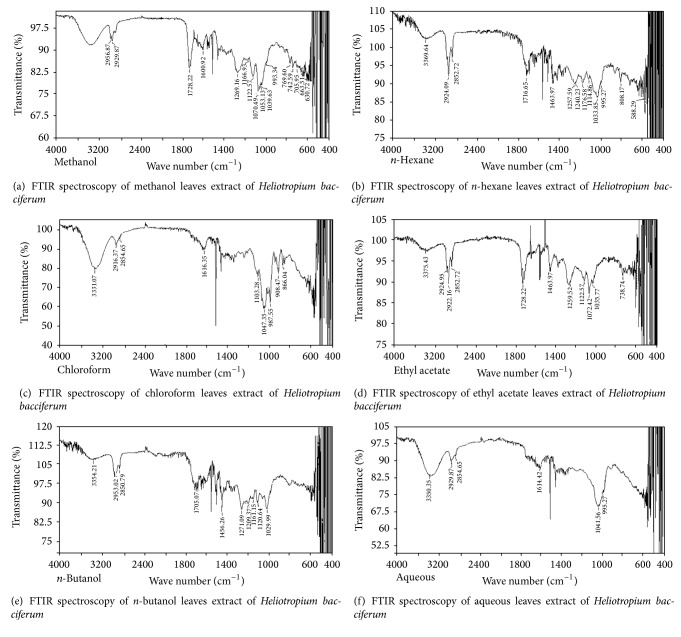
FTIR spectroscopy of flower (a) methanol, (b)* n*-hexane, (c) chloroform, (d) ethyl acetate, (e)* n*-butanol, and (f) aqueous extracts of* Heliotropium bacciferum.*

Compounds functional groups were examined by Fourier transform infrared spectroscopic studies by their peak values (cm^−1^). Alkanes, alkenes, alkyl halides, amines, carboxylic acids, amides, esters, alcohols, phenols, nitrocompounds, and aromatic compounds were identified. Amines, amides, alcohols, phenols, and alkanes showed main peaks at 3370, 3331, 2957, 2924, and 2930 cm^−1^ (Figure 1). Different intensity peaks were identified for carboxylic acids (1717 cm^−1^ and 1705 cm^−1^), unsaturated esters (1728 cm^−1^), 1° amine (1616 cm^−1^), aromatic compounds (1464 cm^−1^), aromatic amines (1258 cm^−1^), aliphatic amines (1123 cm^−1^), 1072, alkenes (1030 cm^−1^ and 1036 cm^−1^), and alkyl halides (808 cm^−1^ and 866 cm^−1^) (Tables 4 [Table tab5] [Table tab6] [Table tab7] [Table tab8]–9).

### 3.5. Thin Layer Chromatography (TLC) Bioautography

Thin layer chromatography bioautography technique is used for bioactive components isolation on TLC plates which link these compounds with the biological activities. Bioautography name is used due to the connection of TLC with the biological activities especially antimicrobial activities. Bands visualization was accomplished by ultraviolet light at 305 and 368 nm. *R*_*f*_ values, inhibition of microorganism's growth, and the active bands were found out by TLC bioautography (Tables 10 and 11). Highest *R*_*f*_ values were recorded against different bacterial strains by plant leaves ethyl acetate (0.93), chloroform (0.76), and methanol (0.85) extracts at EMW, CEF, and BEA solvent systems. Aqueous and* n*-butanol leaves extracts were found inactive at EMW and CEF solvent systems. Plant flower methanol (0.84) and ethyl acetate (0.70) extracts revealed highest *R*_*f*_ values at EMW and BEA solvent systems. Aqueous and* n*-hexane extracts of plant stem were found inactive, while methanol (0.84), ethyl acetate (0.70), and chloroform (0.72) showed high *R*_*f*_ values against different bacterial species. Inhibition areas and *R*_*f*_ values comparison was carried out against the spots on the reference plate. Active compounds in plant leaves extracts against* E. coli* (0.85, 0.93, and 0.76),* S. aureus* (0.89, 0.76, and 0.52), and* B. cereus* (0.73, 0.61, and 0.52) were found at different *R*_*f*_ values by EMW, CEF, and BEA solvent systems. Flower extracts active compounds against* P. aeruginosa* and* K. pneumoniae* were found at 0.84, 0.72, 0.53, and 0.49 *R*_*f*_ values. Stem extracts active compounds were also found at different *R*_*f*_ values against* P. mirabilis* and* E. carotovora* (Table 10). Various *R*_*f*_ values were found to be against different fungal species by plant leaves, flowers, and stem extracts at diverse solvent systems. Leaves ethyl acetate (0.82 and 0.72) and methanol (0.72 and 0.62) extracts were found active and revealed highest *R*_*f*_values against* A. niger* and* A. flavus*. Methanol (0.53, 0.64, and 0.72) and ethyl acetate (0.60 and 0.44) extracts of plant flower revealed significant *R*_*f*_ values against* A. parasiticus* and* A. oryzae.* Stem chloroform (0.51), ethyl acetate (0.73 and 0.69), and aqueous (0.39) extract showed high *R*_*f*_ values against* A. fumigatus*. Inhibition areas and *R*_*f*_ values were compared with standard plate. Plant leaves, flowers, and stem active compounds were found at various *R*_*f*_ values against several fungal strains (Table 11).

## 4. Discussion

It is evident from the results of the present study that* Heliotropium bacciferum* is therapeutically imperative plant species. Prominent and significant antibacterial and antifungal activities were recorded by various parts of plant extracts against antibiotic resistant microbes. Previously reported data on the antimicrobial activities of aerial parts of* Heliotropium bacciferum* revealed that* n*-hexane and ethyl acetate extracts have significant antimicrobial effects (zone of inhibition ranged from 18–30 mm) against different microorganisms. Plant fractions were active against* Salmonella typhi*,* Pseudomonas aeruginosa*,* Escherichia coli*, and* Klebsiella pneumoniae*. Crude, aqueous, and* n-*butanol (2 mg/mL) extracts inhibited the growth of* Trichoderma longibrachiatum*, while it was inhibited by 1 mg/mL* n-*hexane and ethyl acetate extracts of the plant.* Aspergillus flavus* were inhibited by crude, ethyl acetate, and* n-*butanol (1 mg/mL) fractions of plant extract [12]. Plants and their extracts used in disease healing date back to 460–370 BC, when Hippocrates used drugs obtained from plants for remedial purposes [15]. In the present study, the results signified that the plant extracts inhibited the growth of different microorganisms, therefore showing that the plant extracts contained substances which can inhibit the growth of different microbes. Different researchers have also shown that the plant extracts inhibit the growth of diverse microorganisms [16]. A study reported that plant extracts antibacterial potential is mainly due to the presence of different phytochemicals [17]. Many observed that the antimicrobial effects of plant extracts are due to the presence of various secondary metabolites [16]. Traditionally, plant extracts are used in the treatment of sore, boils in the ear, wound healing, diarrhea, and control dysentery [18]. Plant extracts divulged noteworthy activities against* Pseudomonas aeruginosa* and* Staphylococcus aureus*, which explore their use in the cure of bores, open wounds, and sores [19].* Staphylococcus aureus* infections healing has become problematic as it has developed several mechanisms to become resistant to nearly all notorious antibiotics [20, 21]. Antibacterial activity of plant extracts against* Escherichia coli* justifies their use in the treatment of diarrhea and dysentery.* Escherichia coli* is the major reason of diarrhea and in humans other diverse diarrhoeagenic infections [22]. Phenolic compounds due to its peroxidation inhibition and scavenging of oxygen radical are vital for antioxidant activity. These compounds are significant for the treatment of cancer, cardiovascular disorders, inflammatory diseases, and aging [23]. Some phytochemicals such as flavonoids, anthocyanin, catechin, coumarin, isoflavones, flavones, isocatechin, and lignans are responsible for radical scavenging potential [24]. Previous study reported the presence of different bioactive constituents such as alkanes, carboxylic acids, aldehydes, ethers, alcohols, ketones, and amindes by Fourier transform infrared spectroscopic study [10]. Fourier transform infrared spectroscopy has been revealed to be a significant mean for classification and differentiation of intimately relevant microbial species, plants, and other diverse organisms [25–27]. Fourier transform infrared spectrophotometric assessment showed that alkyl halides and alkanes prevalent in plant extracts contained elevated number of functional groups, which were found more significant against* Staphylococcus aureus*,* Candida albicans*, and* Escherichia coli* [28]. Chemical constituents of plants could yield pharmaceutically significant drugs [29].

## 5. Conclusion

Results of the present study revealed noteworthy antimicrobial and antioxidant activities of different parts of* Heliotropium bacciferum*. Plant extracts have immense potential as antibacterial and antifungal compounds against antibiotic resistant microorganisms. Therefore, they can be used in the cure of infectious diseases caused by resistant microorganisms. Alkanes, alkenes, alkyl halides, amines, carboxylic acids, amides, esters, alcohols, phenols, nitrocompounds, and aromatic compounds were identified by FTIR spectroscopic analysis. The results of this evaluation give evidence that* Heliotropium bacciferum* might be a promising source of phytocompounds which can be isolated and analyzed for pharmacological activities,* in vitro* and* in vivo* bioassays.

## Figures and Tables

**Table 1:** Antibacterial activity of all extracts of *Heliotropium bacciferum*.

Plant parts	Plant extracts (*µ*g)	Zone of inhibition (mm) ± standard error mean							St. dev^*α*^
		Tested bacterial strains							
		EC	SA	BC	PA	KP	PM	ECA	
Leaves	Methanol	13 ± 0.32	16 ± 0.51	15 ± 0.39	16 ± 0.44	18 ± 0.46	12 ± 0.62	17 ± 0.35	2.138
	*n-*Hexane	19 ± 0.46	17 ± 0.34	14 ± 0.53	17 ± 0.58	20 ± 0.71	14 ± 0.34	15 ± 0.34	2.370
	Chloroform	14 ± 0.29	R	12 ± 0.62	16 ± 0.37	17 ± 0.73	13 ± 0.45	R	3.988
	Ethyl acetate	18 ± 0.65	19 ± 0.53	13 ± 0.53	15 ± 0.53	21 ± 0.69	12 ± 0.56	14 ± 0.67	3.367
	*n-*Butanol	13 ± 0.36	R	11 ± 0.38	14 ± 0.75	10 ± 0.28	9 ± 0.44	R	2.734
	Aqueous	10 ± 0.72	R	R	R	11 ± 0.39	10 ± 0.29	13 ± 0.46	2.360

Flowers	Methanol	15 ± 0.53	13 ± 0.33	12 ± 0.27	17 ± 0.54	18 ± 0.73	14 ± 0.55	12 ± 0.43	2.370
	*n-*Hexane	17 ± 0.46	19 ± 0.76	15 ± 0.63	18 ± 0.66	19 ± 0.75	13 ± 0.34	15 ± 0.55	2.299
	Chloroform	13 ± 0.42	10 ± 0.64	14 ± 0.52	13 ± 0.43	16 ± 0.64	12 ± 0.47	R	2.911
	Ethyl acetate	16 ± 0.64	20 ± 0.74	15 ± 0.62	13 ± 0.67	19 ± 0.48	14 ± 0.69	12 ± 0.31	2.992
	*n-*Butanol	14 ± 0.34	11 ± 0.54	R	15 ± 0.27	13 ± 0.46	R	11 ± 0.36	3.185
	Aqueous	12 ± 0.45	10 ± 0.27	R	R	10 ± 0.52	9 ± 0.24	13 ± 0.54	2.289

Stem	Methanol	12 ± 0.46	15 ± 0.63	13 ± 0.74	14 ± 0.58	16 ± 0.62	15 ± 0.53	13 ± 0.37	1.414
	*n-*Hexane	16 ± 0.57	15 ± 0.48	18 ± 0.60	16 ± 0.47	14 ± 0.37	14 ± 0.36	11 ± 0.46	2.193
	Chloroform	15 ± 0.53	R	13 ± 0.53	14 ± 0.64	17 ± 0.56	10 ± 0.28	10 ± 0.33	3.450
	Ethyl acetate	13 ± 0.60	16 ± 0.53	17 ± 0.45	12 ± 0.32	16 ± 0.41	15 ± 0.29	15 ± 0.40	1.773
	*n-*Butanol	11 ± 0.43	15 ± 0.52	13 ± 0.28	11 ± 0.43	15 ± 0.64	R	R	3.352
	Aqueous	10 ± 0.29	R	R	R	13 ± 0.42	10 ± 0.29	11 ± 0.26	2.360

*Control*	Ampicillin	27 ± 0.65	25 ± 0.58	23 ± 0.46	25 ± 0.59	29 ± 0.84	26 ± 0.63	22 ± 0.67	2.463

Zone of inhibition (mm) showing sensitivity. R: no inhibition zone (resistance); ampicillin: 8 *µ*g; *α*: standard deviations; EC: *Escherichia coli*; SA: *Staphylococcus aureus*; BC: *Bacillus cereus*; PA: *Pseudomonas aeruginosa*; KP: *Klebsiella pneumoniae*; PM: *Proteus mirabilis*; and ECA:* Erwinia carotovora*.

**Table 2:** Antifungal activity of all extracts of *Heliotropium bacciferum*.

Plant parts	Plant extracts (*µ*g)	Zone of inhibition (mm) ± standard error mean					St. dev^*α*^
		Tested fungal strains					
		AN	AFL	AP	AO	AF	
Leaves	Methanol	17 ± 0.44	15 ± 0.38	14 ± 0.51	11 ± 0.54	R	3.899
	*n-*Hexane	14 ± 0.52	17 ± 0.67	11 ± 0.70	16 ± 0.68	13 ± 0.53	2.387
	Chloroform	12 ± 0.28	13 ± 0.53	R	16 ± 0.45	15 ± 0.49	3.507
	Ethyl acetate	15 ± 0.43	17 ± 0.32	12 ± 0.46	17 ± 0.83	12 ± 0.61	2.510
	*n-*Butanol	11 ± 0.43	14 ± 0.51	R	15 ± 0.57	10 ± 0.29	3.209
	Aqueous	10 ± 0.27	R	9 ± 0.28	11 ± 0.25	R	1.789

Flowers	Methanol	14 ± 0.25	17 ± 0.63	12 ± 0.25	14 ± 0.60	11 ± 0.20	2.302
	*n-*Hexane	17 ± 0.63	15 ± 0.48	13 ± 0.40	12 ± 0.27	15 ± 0.58	2.191
	Chloroform	11 ± 0.26	14 ± 0.46	R	13 ± 0.35	11 ± 0.28	2.683
	Ethyl acetate	16 ± 0.59	15 ± 0.59	11 ± 0.35	15 ± 0.44	10 ± 0.45	2.702
	*n-*Butanol	13 ± 0.47	11 ± 0.23	R	16 ± 0.39	14 ± 0.23	3.421
	Aqueous	R	12 ± 0.38	10 ± 0.31	9 ± 0.22	R	2.121

Stem	Methanol	16 ± 0.54	13 ± 0.42	15 ± 0.37	13 ± 0.48	15 ± 0.51	1.342
	*n-*Hexane	13 ± 0.35	11 ± 0.31	16 ± 0.63	17 ± 0.54	12 ± 0.33	2.588
	Chloroform	15 ± 0.54	16 ± 0.56	R	12 ± 0.27	14 ± 0.37	3.564
	Ethyl acetate	12 ± 0.26	18 ± 0.50	14 ± 0.28	15 ± 0.55	16 ± 0.54	2.236
	*n-*Butanol	15 ± 0.34	R	R	12 ± 0.26	11 ± 0.28	3.435
	Aqueous	R	R	12 ± 0.22	10 ± 0.25	R	2.302

*Control*	Clotrimazole	24 ± 0.82	22 ± 0.87	20 ± 0.78	26 ± 0.79	22 ± 0.68	2.280

Zone of Inhibition (mm) showing sensitivity. R: no inhibition zone (resistance); clotrimazole: 8 *µ*g; *α*: standard deviations; AN: *Aspergillus niger*; AFL: *Aspergillus flavus*; AP: *Aspergillus parasiticus*; AO: *Aspergillus oryzae*; and AF: *Aspergillus fumigatus*.

**Table 3:** Antioxidant activities of flower extracts of *Heliotropium bacciferum*.

Plant extracts	Quantity (*µ*g/mL), mean value ± standard deviation				
	Antioxidant activity (%)				
	25	75	125	175	225
Crude	70.73 ± 0.58	73.55 ± 0.79	75.12 ± 0.66	81.72 ± 0.82	85.75 ± 0.52
*n-*Hexane	65.47 ± 0.67	68.49 ± 0.48	73.86 ± 0.83	78.56 ± 0.71	80.69 ± 0.80
Chloroform	70.54 ± 0.49	74.35 ± 0.54	76.26 ± 0.70	82.78 ± 0.68	86.34 ± 0.75
Ethyl Acetate	71.27 ± 0.35	75.98 ± 0.48	77.27 ± 0.64	83.34 ± 0.59	88.27 ± 0.81
*n-*Butanol	66.33 ± 0.51	70.74 ± 0.62	74.47 ± 0.66	78.54 ± 0.82	81.14 ± 0.94
Aqueous	46.19 ± 0.37	58.82 ± 0.42	65.63 ± 0.73	69.33 ± 0.60	72.36 ± 0.84
*Control*	75.12 ± 0.42	80.92 ± 0.56	86.35 ± 0.68	88.32 ± 0.52	91.58 ± 0.83

**Table 4:** FTIR spectra of flower methanol extract of *Heliotropium bacciferum*.

S. number	Peak values/wave number (cm^−1^)	Type of bond	Functional group
1	2957	C–H stretch	Alkanes
2	2930	C–H stretch	Alkanes
3	1728	C=O stretch	Esters, saturated aliphatic
4	1600	C–C stretch in ring 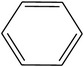	Aromatic compounds
5	1269	C–H wag (–CH_2_X)	Alkyl halides
6	1167	C–N stretch	Aliphatic amines
7	1123	C–N stretch	Aliphatic amines
8	1070	C–N stretch	Aliphatic amines
9	993	=C–H bend	Alkenes
10	770	C–Cl stretch	Alkyl halides
11	743	C–Cl stretch	Alkyl halides
12	707	C–H rock	Alkanes
13	663	–C=C–H, C–H bend	Alkynes
14	631	C–Br stretch	Alkyl halides

**Table 5 tab5:** FTIR spectra of flower *n*-hexane extract of *Heliotropium bacciferum*.

S. number	Peak values/wave number (cm^−1^)	Type of bond	Functional group
1	3370	N–H stretch	1°, 2° amines, amides
2	2924	C–H stretch	Alkanes
3	2853	C–H stretch	Alkanes
4	1717	C=O stretch	Carboxylic acids
5	1464	C–C stretch in ring 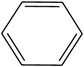	Aromatic compounds
6	1258	C–N stretch 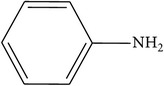	Aromatic amines
7	1240	C–N stretch	Aromatic amines
8	1177	C–N stretch	Aliphatic amines
9	1115	C–N stretch	Aliphatic amines
10	1034	=C–H bend	Alkenes
11	996	O–H bend	Carboxylic Acids
12	808	C–Cl stretch	Alkyl halides
13	588	C–Br tretch	Alkyl halides

**Table 6 tab6:** FTIR spectra of flower chloroform extract of *Heliotropium bacciferum*.

S. number	Peak values/wave number (cm^−1^)	Type of bond	Functional group
1	3331	O–H, H–bonded 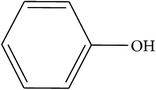	Alcohols, phenols
2	2916	C–H stretch	Alkanes
3	2855	C–H stretch	Alkanes
4	1616	N–H bend	1° amines
5	1103	C–N stretch	Aliphatic amines
6	1047	=C–H bend	Alkenes
7	987	=C–H bend	Alkenes
8	908	N–H wag	1°, 2° amines
9	866	C–Cl stretch	Alkyl halides

**Table 7 tab7:** FTIR spectra of flower ethyl acetate extract of *Heliotropium bacciferum*.

S. number	Peak values/wave number (cm^−1^)	Type of bond	Functional group
1	3375	O–H, H–bonded 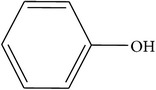	Alcohols, phenols
2	2955	C–H stretch	Alkanes
3	2922	C–H stretch	Alkanes
5	1728	C=O stretch	*α*, *β*-unsaturated esters
6	1464	N–O symmetric stretch	Nitrocompounds
7	1259	C–N stretch 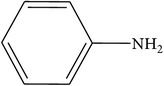	Aromatic amines
8	1123	C–N stretch	Aliphatic amines
9	1072	C–N stretch	Aliphatic amines
10	1036	=C–H bend	Alkenes
11	739	C–Cl stretch	Alkyl halides

**Table 8 tab8:** FTIR spectra of flower *n*-butanol extract of *Heliotropium bacciferum*.

S. number	Peak values/wave number (cm^−1^)	Type of bond	Functional group
1	3354	O–H, H–bonded 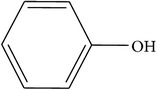	Alcohols, phenols
2	2953	C–H stretch	Alkanes
3	2851	C–H stretch	Alkanes
4	1705	C=O stretch	Carboxylic acid
5	1466	C–C stretch in ring 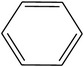	Aromatic compounds
6	1271	C–N stretch 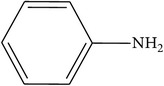	Aromatic amines
7	1209	C–N stretch	Aliphatic amines
8	1161	C–H wag (–CH_2_X)	Alkyl halides
9	1121	C–N stretch	Aliphatic amines
10	1030	=C–H bend	Alkenes

**Table 9:** FTIR spectra of flower aqueous extract of *Heliotropium bacciferum*.

S. number	Peak values/wave number (cm^−1^)	Type of bond	Functional group
1	3341	O–H, H–bonded 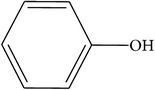	Alcohols, phenols
2	2924	C–H stretch	Alkanes
3	2853	C–H stretch	Alkanes
4	1732	C=O stretch	Carbonyl (aldehydes)
5	1456	N–O asymmetric stretch	Nitrocompounds
6	1049	C–N stretch	Aliphatic amines
7	908	O–H bend	Carboxylic acids

**Table 10:** Growth Inhibition on bioautographic TLC plates by *Heliotropium bacciferum* leaves, flowers, and stem extracts against different bacterial strains.

Plant parts	Bacterial species	Solvent system	Extracts	*R*_*f*_ values	Inhibition	Active bands
Leaves	*Escherichia coli*	EMW	Methanol	0.62, 0.85	+++	2
			*n-*Hexane	0.24	+	1
			Chloroform	0.76	++	1
			Ethyl acetate	0.93	++	1
			*n-*Butanol	0.35, 0.54	+	2
			Aqueous	−	−	−
	*Staphylococcus aureus*	CEF	Methanol	0.89	+++	1
			*n-*Hexane	0.52	++	1
			Chloroform	0.55	+	1
			Ethyl acetate	0.76	++	1
			*n-*Butanol	−	−	−
			Aqueous	0.23, 0.31	+	2
	*Bacillus cereus*	BEA	Methanol	0.52	++	1
			*n-*Hexane	0.47	+	1
			Chloroform	0.61	++	1
			Ethyl acetate	0.73	+++	1
			*n-*Butanol	0.49	+	1
			Aqueous	0.56	++	1

Flowers	*Pseudomonas aeruginosa*	EMW	Methanol	0.84	++	1
			*n-*Hexane	0.67	+++	1
			Chloroform	0.49	+	1
			Ethyl acetate	0.72, 0.64	+++	2
			*n-*Butanol	0.55	+	1
			Aqueous	0.39	+	1
	*Klebsiella pneumoniae*	CEF	Methanol	0.66	+	1
			*n-*Hexane	0.53, 0.43, 0.49	++	3
			Chloroform	0.71	++	1
			Ethyl acetate	0.48	+	1
			*n-*Butanol	0.67	++	1
			Aqueous	−	−	−

Stem	*Proteus mirabilis*	BEA	Methanol	0.84	+++	1
			*n-*Hexane	−	−	−
			Chloroform	0.58	++	1
			Ethyl acetate	0.70	+++	1
			*n-*Butanol	0.28, 0.32	+	2
			Aqueous	0.42	+	1
	*Erwinia carotovora*	EMW	Methanol	0.68	++	1
			*n-*Hexane	0.49	+	1
			Chloroform	0.72, 0.69	++	2
			Ethyl acetate	0.88	+++	1
			*n-*Butanol	0.41	+	1
			Aqueous	−	−	−

*R*_*f*_: retardation factor; −: inactive component; +: small inhibition; ++: moderate inhibition; and +++: high inhibition.

**Table 11:** Growth Inhibition on bioautographic TLC plates by *Heliotropium bacciferum* leaves, flowers, and stem extracts against different fungal strains.

Plant parts	Fungal species	Solvent system	Extracts	*R*_*f*_ values	Inhibition	Active bands
Leaves	*Aspergillus niger*	EMW	Methanol	0.62	+	1
			*n-*Hexane	0.44	++	1
			Chloroform	0.38, 0.41	+	2
			Ethyl acetate	0.82	+++	1
			*n-*Butanol	0.34, 0.42	++	2
			Aqueous	0.27	+	1
	*Aspergillus flavus*	CEF	Methanol	0.72	++	1
			*n-*Hexane	−	−	−
			Chloroform	0.63	++	2
			Ethyl acetate	0.70, 0.66	++	2
			*n-*Butanol	0.43	+	1
			Aqueous	0.32	++	1

Flowers	*Aspergillus parasiticus*	BEA	Methanol	0.53	+++	1
			*n-*Hexane	0.47, 0.37, 0.41	++	3
			Chloroform	−	−	−
			Ethyl acetate	0.60	+	1
			*n-*Butanol	0.43	++	1
			Aqueous	0.38	+	1
	*Aspergillus oryzae*	EMW	Methanol	0.64, 0.72	+++	2
			*n-*Hexane	0.55	++	1
			Chloroform	0.42	+	1
			Ethyl acetate	0.44	++	1
			*n-*Butanol	0.35	+	1
			Aqueous	0.54	++	1

Stem	*Aspergillus fumigatus*	CEF	Methanol	0.68	++	1
			*n-*Hexane	0.42	+	1
			Chloroform	0.51	++	1
			Ethyl acetate	0.73, 0.69	+++	2
			*n-*Butanol	−	−	−
			Aqueous	0.39	++	1

*R*_*f*_: retardation factor; −: inactive component; +: small inhibition; ++: moderate inhibition; and +++: high inhibition.

## References

[1] Mahesh B., Satish S. (2008). Antimicrobial activity of some important medicinal plants against plants and human pathogens. *World Journal of Agricultural Sciences*.

[2] Abeysinghe P. D. (2012). Antibacterial activity of aqueous and ethanol extracts of mangrove species collected from Southern Sri Lanka. *Asian Journal of Pharmaceutical and Biological Research*.

[3] Chandra R., Dwivedi V., Shivam K., Jha A. K. (2011). Detection of antimicrobial activity of *Oscimum sanctum* (Tulsi) & *Trigonella foenum* graecum (Methi) against some selected bacterial & fungal strains. *Research Journal of Pharmaceutical, Biological and Chemical Sciences*.

[4] Iwalokun B. A., Ogunledun A., Ogbolu D. O., Bamiro S. B., Jimi-Omojola J. (2004). *In vitro* antimicrobial properties of aqueous garlic extract against multidrug-resistant bacteria and *Candida* species from Nigeria. *Journal of Medicinal Food*.

[5] Tsao S.-M., Yin M.-C. (2001). *In vitro* antimicrobial activity of four diallyl sulphides occurring naturally in garlic and Chinese leek oils. *Journal of Medical Microbiology*.

[6] Nascimento G. G. F., Locatelli J., Freitas P. C., Silva G. L. (2000). Antibacterial activity of plant extracts and phytochemicals on antibiotic-resistant bacteria. *Brazilian Journal of Microbiology*.

[7] Maleki S., Seyyednejad S. M., Damabi N. M., Motamedi H. (2008). Antibacterial activity of the fruits of Iranian *Torilis leptophylla* against some clinical pathogens. *Pakistan Journal of Biological Sciences*.

[8] Ali S. I., Nasir Y. J. (1983). *Flora of Pakistan*.

[9] Iqbal K., Nawaz S. A., Malik A. (2005). Isolation and lipoxygenase-inhibition studies of phenolic constituents from *Ehretia obtusifolia*. *Chemistry and Biodiversity*.

[10] Ahmad S., Ahmad S., Bibi A. (2014). Phytochemical analysis, antioxidant activity, fatty acids composition, and functional group analysis of *Heliotropium bacciferum*. *Scientific World Journal*.

[11] Murugesh K., Yeligar V., Dash D. K., Sengupta P., Maiti B. C., Maity T. K. (2006). Antidiabetic, antioxidant and antihyperlipidemic status of *Heliotropium zeylanicum* extract on streptozotocin-induced diabetes in rats. *Biological and Pharmaceutical Bulletin*.

[12] Ahmad S., Ahmad S., Bibi I. (2015). Antibacterial and antifungal activities of the extract and fractions of aerial parts of *Heliotropium bacciferum*. *African Journal of Traditional, Complementary and Alternative Medicines*.

[13] Rehman A., Fatima F., Ullah H. (2013). Antibacterial study of medicinal plant *Trigonella foenum*. *International Journal of Basic Medical Sciences and Pharmacy*.

[14] Ishaq M. S., Hussain M. M., Siddique Afridi M. (2014). *In vitro* phytochemical, antibacterial, and antifungal activities of leaf, stem, and root extracts of *Adiantum capillus veneris*. *The Scientific World Journal*.

[15] Soforowa E. A. (1982). *Medicinal Plants and Traditional Medicine in Africa*.

[16] Nweze E. I., Okafor J. I., Njoku O. (2004). Antimicrobial activities of methanolic extracts of *Trema guineensis* (Schumm and Thorn) and *Morinda lucida* Benth used in Nigerian Herbal Medicinal Practice. *Bio-Research*.

[17] Draughon F. A. (2004). Use of botanicals as bio preservatives in foods. *Food Technology*.

[18] Igoli J. O., Ogaji T. A., Tor A., Igoli N. P. (2005). FTraditional medicine practice amongst the igede people of Nigeria part II. *African Journal of Traditional, Complementary and Alternative Medicine*.

[19] Braude A. I. (1982). *Microbiology*.

[20] Lowy F. D. (1998). Medical progress: *Staphylococcus aureus* infections. *The New England Journal of Medicine*.

[21] Morell E. A., Balkin D. M. (2010). Methicillin-resistant *Staphylococcus aureus*: a pervasive pathogen highlights the need for new antimicrobial development. *Yale Journal of Biology and Medicine*.

[22] Adams M. R., Moss M. O. (1999). *Food Microbiology*.

[23] Cioffi G., D'Auria M., Braca A. (2002). Antioxidant and free-radical scavenging activity of constituents of the leaves of *Tachigalia paniculata*. *Journal of Natural Products*.

[24] Aqil F., Ahmad I., Mehmood Z. (2006). Antioxidant and free radical scavenging properties of twelve traditionally used Indian medicinal plants. *Turkish Journal of Biology*.

[25] Das A. J., Khawas P., Miyaji T., Deka S. C. (2016). Phytochemical constituents, attenuated total reflectance fourier transform infrared analysis and antimicrobial activity of four plant leaves used for preparing rice beer in Assam, India. *International Journal of Food Properties*.

[26] Lamprell H., Mazerolles G., Kodjo A., Chamba J. F., Noël Y., Beuvier E. (2006). Discrimination of *Staphylococcus aureus* strains from different species of *Staphylococcus* using Fourier transform infrared (FTIR) spectroscopy. *International Journal of Food Microbiology*.

[27] Rebuffo C. A., Schmitt J., Wenning M., Von Stetten F., Scherer S. (2006). Reliable and rapid identification of Listeria monocytogenes and Listeria species by artificial neural network-based fourier transform infrared spectroscopy. *Applied and Environmental Microbiology*.

[28] Janakiraman N., Sahaya Sathish S., Johnson M. (2011). UV-VIS and FTIR spectroscopic studies on *Peristrophe bicalyculata* (RETZ.) Nees. *Asian Journal of Pharmaceutical and Clinical Research*.

[29] Ullah N., Rehman A., Ahmad S. (2016). Antimicrobial assay and minimum inhibitory concentration values of *Cistanche tubulosa*. *International Journal of Current Microbiology and Applied Sciences*.

